# Bayesian network models to assess antimicrobial resistance patterns of *Streptococcus suis* isolated from swine production systems in the United States between 2014–2021

**DOI:** 10.1371/journal.pcbi.1014117

**Published:** 2026-03-26

**Authors:** Ruwini Rupasinghe, Brittany L. Morgan Bustamante, Rebecca C. Robbins, Maria J. Clavijo, Beatriz Martínez-López

**Affiliations:** 1 Center for Animal Disease Modeling and Surveillance, Department of Medicine and Epidemiology, Weill School of Veterinary Medicine, University of California, Davis, Davis, California, United States of America; 2 Division of Environmental Health Sciences, University of California, Berkeley, Berkeley, California, United States of America; 3 PIC North America, Hendersonville, Tennessee, United States of America,; 4 Department of Veterinary Diagnostic and Production Animal Medicine, Iowa State University, Ames, Iowa, United States of America; Robert Koch Institut, GERMANY

## Abstract

Multidrug resistance (MDR) is frequently evident in *Streptococcus suis,* generating distinct antimicrobial resistance (AMR) profiles, which limits the effective antimicrobial drug (AMD) options against *S. suis* in pigs and humans. Despite its significance, there is a lack of studies and pertinent methodologies that uncover complex interactions among AMDs and associated resistance patterns. This study aimed to identify associations between phenotypic resistance patterns of *S. suis* isolates from swine production systems in the United States against common AMDs using Bayesian network analysis (BNA). Data from 259 unique *S. suis* isolates collected from 91 farms were included. Phenotypic susceptibility interpretations (resistance *vs* susceptible) of minimum inhibitory concentrations (MICs) were evaluated for 13 commonly used AMDs: ceftiofur (CEF), penicillin (PEN), enrofloxacin (ENR), gentamicin (GEN), neomycin (NEO), spectinomycin (SPC), sulfadimethoxine (SUL), tiamulin (TIA), tilmicosin (TIL), clindamycin (CLN), chlortetracycline (CHL), oxytetracycline (OXY), and tetracycline (TET). BNA was conducted using the R package *bnlearn* to identify joint resistance patterns and estimate conditional dependencies among resistance outcomes. Results revealed a high prevalence of MDR: 248 isolates (95.6%) were resistant to more than one AMD, and 209 isolates (80.7%) were resistant to at least one AMD in three or more classes. The Bayesian network comprised of 11 edges connecting 13 AMD nodes, highlighting statistical dependencies between AMDs resistances. PEN, TIA, and TIL were the most central nodes, with PEN connected to SUL, TIA, GEN, and CEF; TIA to PEN, SPC, TIL, and CLN; and TIL to SUL, TIA, CLN, and OXY. Other associations included CEF–SPC, TET–CLN, CEF–ENR, and OXY–CHL. These relationships implicate systematic dependencies between AMDs and may have resulted from mechanisms like cross-resistance and co-resistance. While these relationships are statistically derived and hypothesis-generating, they underscore the importance of understanding AMR patterns in guiding more effective AMD use. This approach can help prevent overuse, reduce treatment failures, and support AMR mitigation efforts for improved animal and public health outcomes.

## Author summary

*Streptococcus suis* is a bacterial pathogen that affects pigs and can also cause serious infections in humans. Antibiotics are commonly used to prevent and treat *S. suis* infections in pigs, but the growing prevalence of antimicrobial resistance and multidrug resistance has challenged the current control efforts. This presents a significant challenge to both animal and public health. In our study, we aimed to better understand how *S. suis* develops resistance to multiple antibiotics. We analyzed 259 *S. suis* isolates collected from pig farms across the United States, assessing their resistance to 13 commonly used antibiotics. Nearly all isolates were resistant to more than one antibiotic, and many showed resistance across several antibiotic classes. To explore how resistance to one drug might be linked to resistance to others, we used Bayesian network analysis. This approach revealed several statistically significant resistance patterns. Penicillin, a widely used and effective antibiotic, tiamulin, and tilmicosin were linked to resistance to more antibiotics than any other drugs. Our findings highlight the complex nature of antibiotic resistance in *S. suis*. Recognizing these resistance patterns can support more informed treatment decisions, reduce the misuse of antibiotics, and help slow the spread of resistance in animals and people.

## Introduction

*Streptococcus suis* is a zoonotic bacterial pathogen considered one of the most important in swine farming and production [1]. *S. suis* infection causes decreased performance and increased mortality in pigs, resulting in extensive economic losses to the swine industry worldwide [2]. In humans, *S. suis* can cause severe systemic infection and is a common cause of adult meningitis in Southeast Asia [3]. Since there is no effective commercial vaccine against *S. suis* [4], control in pigs relies on antimicrobial drug (AMD) use, which raises concerns for increased risk of antimicrobial resistance (AMR) [1]. The AMD type and use patterns against *S. suis* infection in pigs vary among countries, regions, production systems, and even between farms, which profoundly affects the AMR profiles of *S. suis* [5]. Nevertheless, this bacterium is considered a primary reservoir for AMR, with an increased risk for transmission of antimicrobial resistance genes (ARGs) to other bacterial commensals and pathogens [6].

The discovery of AMDs is one of the best advances in therapeutic medicine in humans and animals. These AMDs either kill target bacteria (bactericidal) or inhibit their growth (bacteriostatic) by interfering with specific processes essential for bacterial growth and/or division or disrupting structures in the bacterial cells [7]. Resistance to such AMDs is a natural process, often occurring by mutations and the acquisition of mobile genetic elements (MGEs) via horizontal gene transfer (HGT) events [8]. However, the current increasing trends in AMR rates are often attributed to the misuse and overuse of AMDs, as well as poor infection prevention and control in both animals and humans. Some bacterial strains have become resistant to many AMDs, the phenomenon broadly called multidrug resistance (MDR), which occurs by accumulating multiple genes, each coding for resistance to a single drug or a single resistance mechanism confers resistance to multiple AMDs (cross-resistance) [9]. MDR is often evident in *S. suis* forming various AMR profiles, which limits the effective AMD choices to treat *S. suis* infection in pigs and humans. Thus, AMR patterns of *S. suis* should be carefully monitored to ensure the efficacy of AMDs and successful resistance control.

MDR in bacteria is a complex but essential notion to be incorporated in AMR epidemiological research, such as risk factor analysis. Analytically, it is similar to multivariate observations with correlations between outcome variables, which is challenging for traditional multivariable regression modeling [10]. One approach when dealing with multiple outcomes is constructing a composite score as a response variable and running a regression analysis [11]. However, a disadvantage of this approach is that we may lose valuable insights by reducing the different outcomes into a single-dimension outcome variable. In recent years, Bayesian Network Analysis (BNA) and Additive Bayesian Networks (ABN) have emerged as valued techniques for uncovering complex interactions between variables in a dataset and assessing patterns and profiles of association between AMR classifications [10,12,13]. While both BNA and ABN use directed acyclic graphs (DAGs) to model conditional dependencies, ABN relies on regression-based parameterization whereas BNA uses fully probabilistic conditional probability tables.

BNAs provide a data-drive representation of the joint probability structure, offering a more complete understanding of the epidemiology of AMR in a study population [10]. Unlike traditional pairwise or regression-based approaches, which assess associations in isolation or under a prespecified model, BNAs evaluate all conditional dependencies simultaneously, allowing detection of relationships that only emerge after accounting for correlated factors [14]. In this study, we seek to add to the growing body of research exploring the AMR of *S. suis* to different AMDs used in swine production. Our objective was to identify a suitable network representing the associations between phenotypic AMR of *S. suis* isolates using passive syndromic surveillance data from large swine production systems in the United States.

## Results

### Summary statistics

MIC interpretations for the AMDs included in the analysis are presented in Table 1. Almost all isolates (n = 257; 99.2%) were resistant to at least one AMD, and 248 (95.6%) were resistant to more than one. Only a few isolates were resistant to CEF (3.9%), GEN (4.2%), and ENR (6.2%), whereas the highest resistance percentages were observed for TET (90%), OXY (85%), CHL (83%), and CLN (78%). A total of 94 different AMR patterns were observed among the isolates (S1 Fig).

**Table 1:** Summary of phenotypic resistance of *S. suis* isolates obtained from clinically ill pigs during 2014-2021 (n = 259), 2014-2018 (n = 119), and 2019-2021 (n = 140). MICs interpreted as intermediate were grouped with susceptible.

Antimicrobial drug class	AMD Interpretation	2014-2021 N (%)	2014-2018 N (%)	2019-2021 N (%)
**Beta*-*lactam**	**Ceftiofur**			
	Resistant	11 (4.2%)	1 (<1%)	10 (7.1%)
	Susceptible	248 (96%)	118 (99%)	130 (93%)
	**Penicillin**			
	Resistant	28 (11%)	9 (7.6%)	19 (14%)
	Susceptible	231 (89%)	110 (92%)	121 (86%)
**Fluoroquinolone**	**Enrofloxacin**			
	Resistant	16 (6.2%)	7 (5.9%)	9 (6.4%)
	Susceptible	243 (94%)	112 (94%)	131 (94%)
**Aminoglycoside**	**Gentamicin**			
	Resistant	11 (4.2%)	4 (3.4%)	7 (5%)
	Susceptible	248 (96%)	115 (97%)	133 (95%)
	**Neomycin**			
	Resistant	177 (68%)	75 (63%)	102 (73%)
	Susceptible	82 (32%)	44 (37%)	38 (27%)
	**Spectinomycin (Aminocyclitol)**			
	Resistant	29 (11%)	11 (9.2%)	18 (13%)
	Susceptible	230 (89%)	108 (91%)	122 (87%)
**Sulfonamide**	**Sulfadimethoxine**			
	Resistant	181 (70%)	79 (66%)	102 (73%)
	Susceptible	78 (30%)	40 (34%)	38 (27%)
**Pleuromutilin**	**Tiamulin**			
	Resistant	50 (19%)	19 (16%)	31 (22%)
	Susceptible	209 (81%)	100 (84%)	109 (78%)
**Macrolide**	**Tilmicosin**			
	Resistant	180 (69%)	80 (67%)	100 (71%)
	Susceptible	79 (31%)	39 (33%)	40 (29%)
**Tetracycline**	**Chlortetracycline**			
	Resistant	95 (83%)	88 (83%)	7 (78%)
	Susceptible	20 (17%)	18 (17%)	2 (22%)
	Unknown	144	13	131
	**Oxytetracycline**			
	Resistant	98 (85%)	91 (86%)	7 (78%)
	Susceptible	17 (15%)	15 (14%)	2 (22%)
	Unknown	144	13	131
	**Tetracycline**			
	Resistant	129 (90%)	10 (77%)	119 (91%)
	Susceptible	15 (10%)	3 (23%)	12 (9.2%)
	Unknown	115	106	9
**Lincosamide**	**Clindamycin**			
	Resistant	196 (78%)	91 (76%)	105 (79%)
	Susceptible	56 (22%)	28 (24%)	28 (21%)
	Unknown	7	0	7

**Fig 1:**
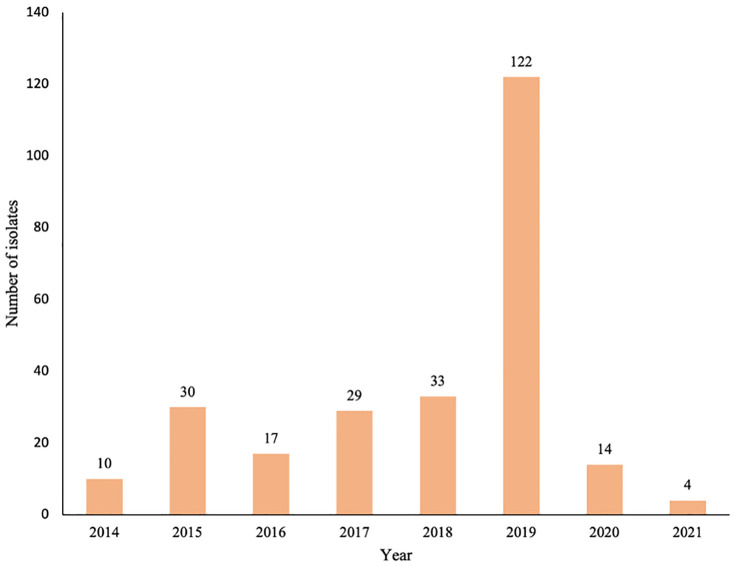
Distribution of sampling of *S. suis* isolates. Temporal distribution of *S. suis* isolates obtained from pigs in each year between 2014-2021 (n = 259).

Most isolates were submitted in 2019 (47%), followed by 2018 (13%). Notably, the higher number of samples submitted in 2019 coincided with an unusually high *S. suis* detection frequency reported by diagnosticians at the Iowa State University Veterinary Diagnostic Laboratory (ISU-VDL). This increase was at least partly attributed to the dissemination of a more pathogenic strain in the region, consistent with a previous report [15]. However, diagnostic submission volumes are influenced by multiple factors, including disease severity, awareness, and diagnostic practices, and may not accurately reflect true population-level incidence. The temporal distribution of isolates obtained for MIC testing is shown in Fig 1.

### Bayesian network analysis

**Fig 2:**
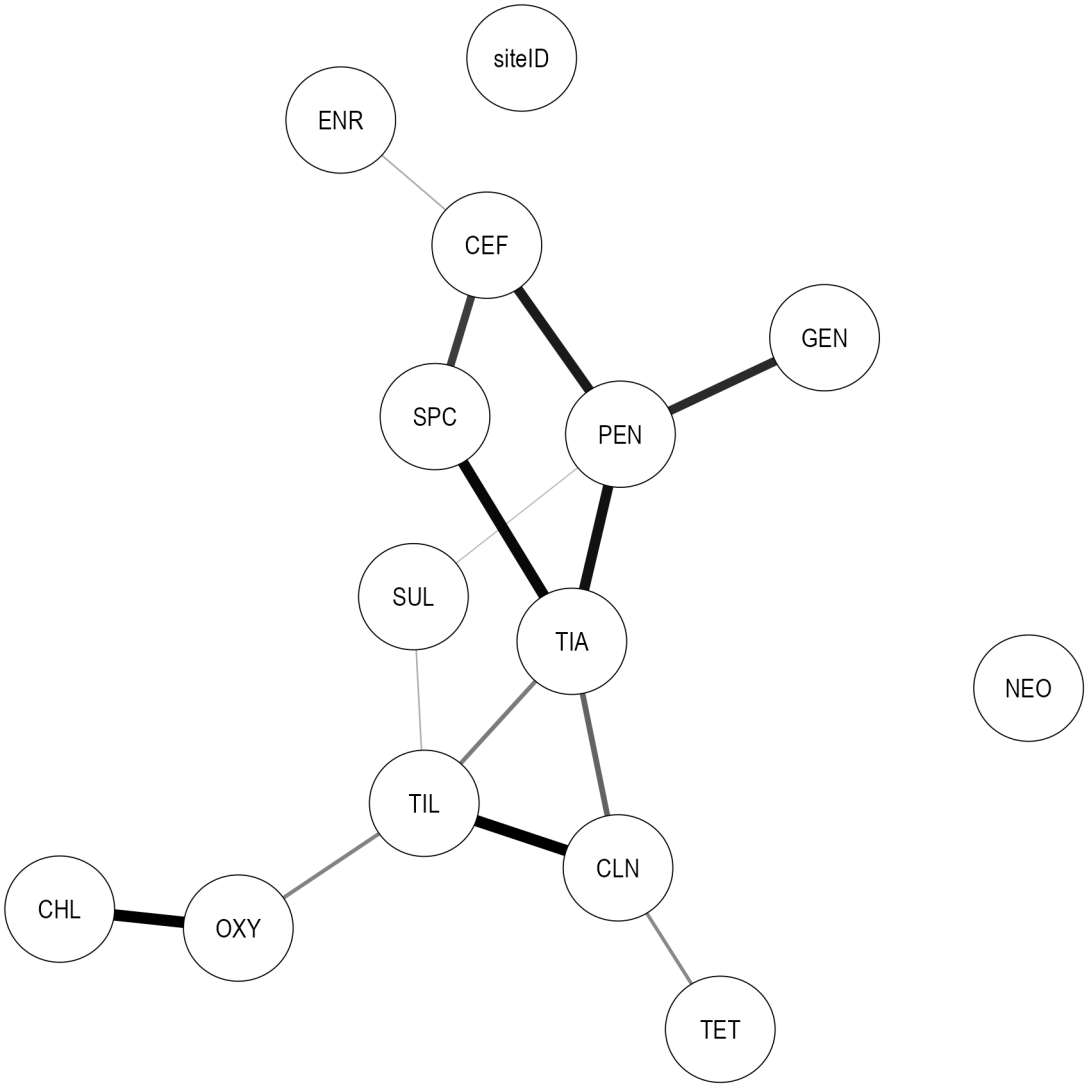
Graph of relationships among phenotypic *S. suis* resistance classifications (2014-2021). Linkages represent associations between resistance classifications (resistant *vs.* susceptible, with intermediate categorized as susceptible) across different AMDs in *S. suis* isolates obtained from swine between 2014-2021. Edge thickness reflects the bootstrap arc strength, defined as the frequency with which each arc was recovered across 10,000 bootstrap networks; only arcs with bootstrap strength >50% are shown. The farm identifier (siteID) is a contextual variable used to account for clustering of isolates by production site. Nodes represent AMDs: Ceftiofur (CEF), enrofloxacin (ENR), gentamicin (GEN), neomycin (NEO), penicillin (PEN), spectinomycin (SPC), sulfadimethoxine (SUL), tiamulin (TIA), tilmicosin (TIL), chlortetracycline (CHL), clindamycin (CLN), oxytetracycline (OXY), and tetracycline (TET).

There was no difference in the network structures built using the Bayes’ and parents’ imputation methods. The parents’ imputation method is performed in topological order and is poorly suited for missing values in nodes that do not have parents, so we present results from the Bayes’ imputation method, which imputes results from their joint posterior distribution conditional on the observed variables. The final bootstrapped model is presented graphically in Fig 2. The network consisted of 11 edges connecting 13 AMD nodes. The following variables were linked together: MIC interpretation for PEN was connected to MIC interpretation for SUL, TIA, GEN, and CEF. Other connections included SUL–TIL, TIA–CLN, TIA–SPC, TIA–PEN, TIA–TIL, CEF–SPC, TET–CLN, CLN–TIL, CEF–ENR, TIL–OXY, and OXY–CHL. These connections indicate statistical dependence between MIC interpretations for these AMDs. That is, whether an *S. suis* isolate was phenotypically susceptible or resistant to one of these AMDs was statistically associated with whether it was phenotypically susceptible or resistant to the connected AMDs.

A complex association among PEN, TIA, SUL, GEN, and CEF was identified, with PEN and TIA each having four edges, among the AMDs with the highest degrees observed in the network. The probability that an isolate showed phenotypic resistance to PEN given that it showed resistance to GEN (R_PEN_|R_GEN_) was 80.6%, compared to just 7.2% if the isolate was susceptible to GEN (R_PEN_|S_GEN_). The relationship between PEN–CEF was similar; the probability of PEN resistance given CEF resistance (R_PEN_|R_CEF_) was 77.4%, compared to 9.2% if the isolate was susceptible to CEF (R_PEN_|S_CEF_). Phenotypic resistance to SUL was associated with both PEN and TIL susceptibility. The probability that an isolate was resistant to SUL given that it was resistant to both PEN and TIL (R_SUL_|R_PEN_ & R_TIL_) was 88.9%. If an isolate was susceptible to either PEN or TIL, the probability of SUL resistance (R_SUL_|S_PEN_ OR S_TIL_) was reduced to 67.8%. Further, if an isolate was susceptible to both PEN and TIL, the probability of SUL resistance (R_SUL_|S_PEN_ & S_TIL_) was 41.6%. The probability of TIA resistance given PEN resistance (R_TIA_|R_PEN_) was 71.2%, and this association was further influenced by CLN, SPC, and TIL susceptibility. For example, if an isolate was resistant to CLN, the probability of TIA resistance given PEN resistance (R_TIA_|R_PEN_ & R_CLN_) remained similar at 71.0%. In contrast, if an isolate was susceptible to CLN, this probability decreased to 53.9% (R_TIA_|R_PEN_ & S_CLN_). Resistance to SPC also did not substantially alter the probability of TIA resistance given PEN resistance (R_TIA_|R_PEN_ & R_SPC_ = 70.5%). Conversely, when isolates were susceptible to both PEN and SPC or to both PEN and CLN, the probability of TIA susceptibility was 91.4% (S_TIA_|S_PEN_ & S_SPC_) and 91.8% (S_TIA_|S_PEN_ & S_CLN_), respectively, indicating strong co-susceptibility patterns among these drugs.

TIL was one of the most central nodes, with four edges connecting it to SUL, TIA, CLN, and OXY. As an example, the association between TIL–SUL–CLN illustrates the complex interdependencies of resistance within the network. The probability of TIL resistance given CLN resistance (R_TIL_|R_CLN_) was 84.2%, compared to 1.3% if an isolate was susceptible to CLN (R_TIL_|S_CLN_). This association was moderately influenced by SUL resistance, increasing to only 88.7% if the isolate was resistant to CLN and SUL (R_TIL_|R_CLN_ & R_SUL_) but reducing to 69.6% if the isolate was resistant to CLN but susceptible to SUL (R_TIL_|R_CLN_ & S_SUL_). Alternatively, the probability that an isolate was resistant to TIL given resistance to SUL (R_TIL_|R_SUL_) was 76.1%. However, this association was highly influenced by CLN resistance. If an isolate was also resistant to CLN, this probability increased to 89.2% (R_TIL_|R_SUL_ & R_CLN_). If an isolate was susceptible to CLN, the probability of TIL resistance given SUL resistance (R_TIL_|R_SUL_ & S_CLN_) was 1.9%.

The MIC interpretation for NEO was completely independent of all other AMDs, as indicated by its separation from the other nodes in the network. ENR was only weakly connected to the network through CEF. There was a statistical association between OXY and CHL, which was slightly influenced by TIL. The probability of OXY resistance given CHL resistance (R_OXY_|R_CHL_) was 99.8%, compared to just 16.1% if the isolate was susceptible to CHL (R_OXY_|S_CHL_). Resistance to TIL slightly increased the probability of OXY resistance given CHL resistance (R_OXY_|R_CHL_ & R_TIL_) to 99.9%. Other influential connections in the network included the probability of SPC resistance given CEF resistance (R_SPC_|R_CEF_) was 56.3%, compared to 10.1% if an isolate was susceptible to CEF (R_SPC_|S_CEF_). The probability of CLN resistance was higher when an isolate was resistant to TET (R_CLN_|R_TET_ = 81.5%) than when an isolate was susceptible to TET (R_CLN_|S_TET_ = 45.0%).

**Fig 3:**
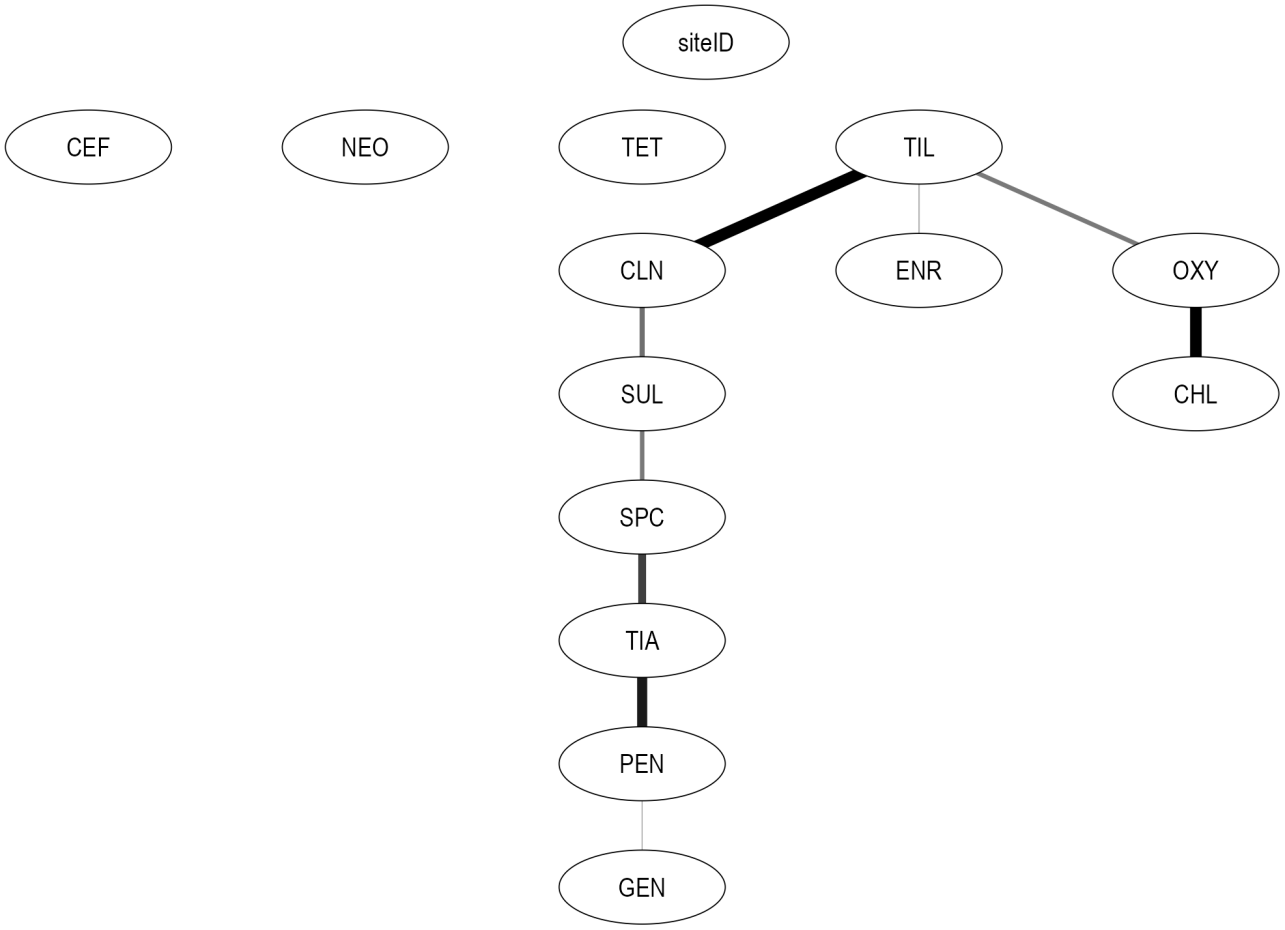
Graph of relationships among phenotypic *S. suis* resistance classifications (2014-2018). Linkages represent associations between resistance classifications (resistant *vs.* susceptible, with intermediate categorized as susceptible) across different AMDs in *S. suis* isolates obtained from swine between 2014-2018. Edge thickness reflects the bootstrap arc strength, defined as the frequency with which each arc was recovered across 10,000 bootstrap networks; only arcs with bootstrap strength >50% are shown. The farm identifier (siteID) is a contextual variable used to account for clustering of isolates by production site. Nodes represent AMDs: Ceftiofur (CEF), enrofloxacin (ENR), gentamicin (GEN), neomycin (NEO), penicillin (PEN), spectinomycin (SPC), sulfadimethoxine (SUL), tiamulin (TIA), tilmicosin (TIL), chlortetracycline (CHL), clindamycin (CLN), oxytetracycline (OXY), and tetracycline (TET).

**Fig 4:**
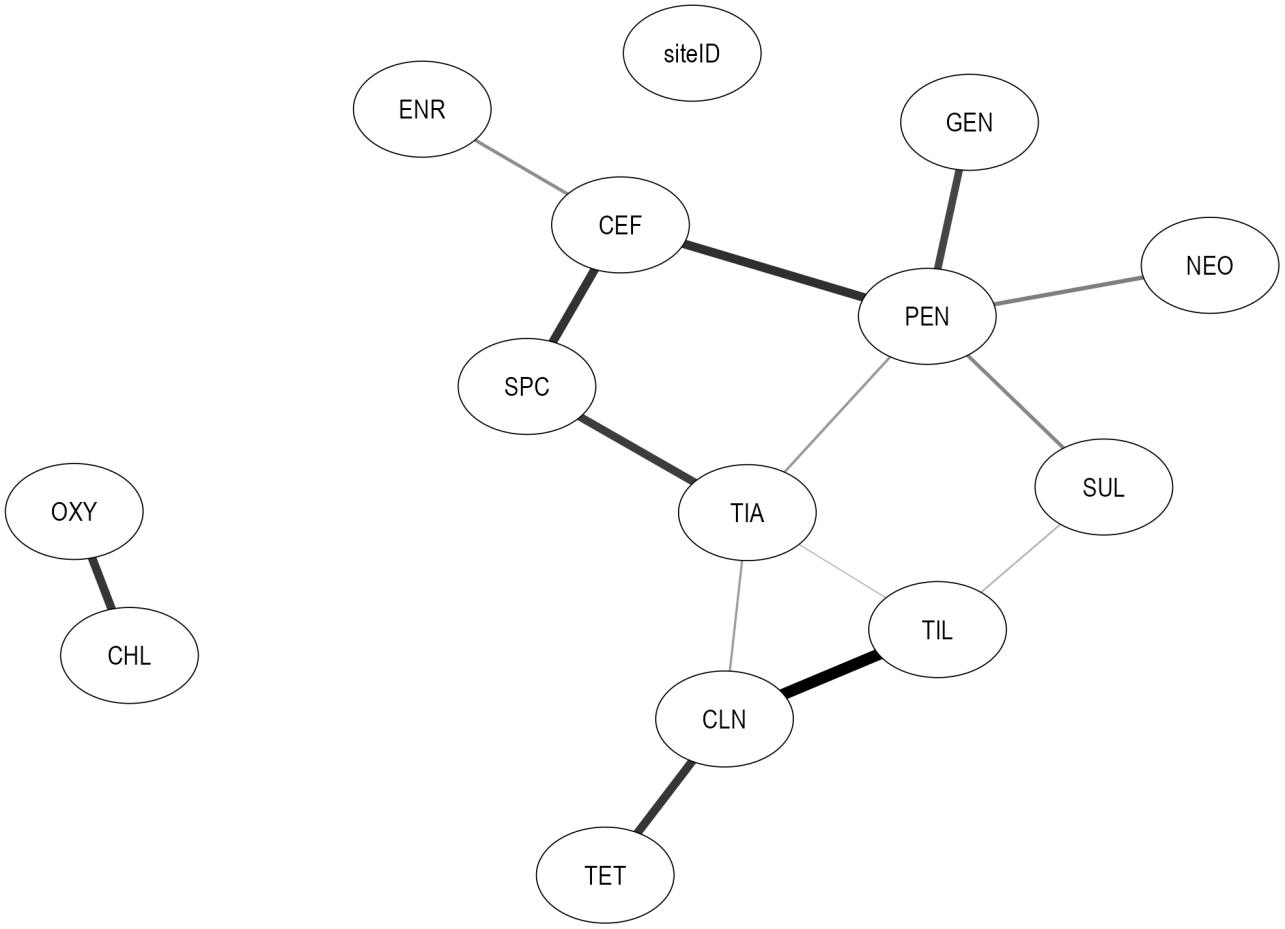
Graph of relationships among phenotypic *S. suis* resistance classifications (2019-2021). Linkages represent associations between resistance classifications (resistant *vs.* susceptible, with intermediate categorized as susceptible) across different AMDs in *S. suis* isolates obtained from swine between 2019-2021. Edge thickness reflects the bootstrap arc strength, defined as the frequency with which each arc was recovered across 10,000 bootstrap networks; only arcs with bootstrap strength >50% are shown. The farm identifier (siteID) is a contextual variable used to account for clustering of isolates by production site. Nodes represent AMDs: Ceftiofur (CEF), enrofloxacin (ENR), gentamicin (GEN), neomycin (NEO), penicillin (PEN), spectinomycin (SPC), sulfadimethoxine (SUL), tiamulin (TIA), tilmicosin (TIL), chlortetracycline (CHL), clindamycin (CLN), oxytetracycline (OXY), and tetracycline (TET).

The two subnetworks highlight differences in joint statistical associations among AMDs under differing data availability conditions (Figs 3 and 4). In the 2014–2018 network, PEN was linked to GEN and TIA, whereas in the 2019–2021 network, PEN was connected to GEN, TIA, NEO, CEF, and SUL. Tetracycline-related associations also differed across subnetworks. In the 2014–2018 network, TET was isolated from other nodes, whereas in the 2019–2021 network, a statistical association was observed between OXY and CHL that was independent of other AMDs, consistent with the limited availability of these data in that period. These findings indicate that the period-specific subnetworks captured associations under differing data availability conditions. Notably, the overall network constructed using data from all years recovered tetracycline-specific associations observed in both period-specific subnetworks, indicating that the overall network integrated tetracycline-related relationships across the study period despite variability in drug-specific reporting. Associations identified in the 2014–2018 network between OXY–TIL, TIL–ENR, SUL–CLN, and SUL–SPC were no longer found in the 2019–2021 network. Further, in 2019–2021, associations were identified between TIA–TIL, TIL–SUL, CEF–ENR, CEF–SPC, CLN–TET, and CLN–TIA. However, these period-specific subnetworks were not designed to assess or compare temporal changes in resistance patterns.

## Discussion

*S. suis* is a widely spread pathobiont among pigs and a neglected emerging zoonotic pathogen that causes systemic diseases in humans. Prophylaxis and treatment of *S. suis* infection in pigs heavily rely on antibiotics, but high AMR and MDR prevalence in *S. suis* has challenged the current control efforts. In our study, almost all the isolates (n = 257; 99.2%) were resistant to at least one AMD with a high prevalence of AMR against NEO, SUL, TIL, CLN, CHL, OXY, and TET (68–90%), which agrees with previous studies [5,16,17]. MDR has various definitions in the medical literature characterizing different resistance patterns found in bacteria; a commonly used definition is non-susceptible to at least one AMD in three or more AMD classes [18,19]. Here, we used a broad definition for MDR, characterizing isolates non-susceptible to more than one AMD, and discussed all the observed statistical associations between AMDs. Nevertheless, we identified substantial MDR within our tested isolates based on both definitions, where 248 (95.6%) isolates were resistant to more than one AMD, and 209 (80.7%) isolates were resistant to at least one AMD in three or more AMD classes.

The AMDs included in this study represent drugs commonly used for therapeutic purposes in U.S. swine medicine and reflect standard susceptibility testing panels used by veterinary diagnostic laboratories. None of these AMDs are approved for use as growth promoters in the United States. Furthermore, the use of AMDs classified as critically important for human medicine is subject to strict regulatory oversight and veterinary prescription requirements under the Veterinary Feed Directive and related FDA policies [20,21]. To characterize AMR profiles and identify statistical associations among phenotypic resistance to these AMDs, we applied BNA, a relatively novel analytical approach [10,12,13], to *S. suis* isolates obtained from clinically ill pigs in the United States.

The observed associations of AMR between drugs within the same class, such as the relationship between OXY and CHL, are not surprising, as these drugs share similar modes of action [22]. Strong conditional dependencies of resistance-resistance (R_AMD-1_|R_AMD-2_) and susceptible-susceptible (S_AMD-1_|S_AMD-2_) outcomes between AMDs (AMD-1 and AMD-2) within the same class, such as those observed between OXY–CHL (e.g., R_OXY_|R_CHL_ = 99.8% and S_OXY_|S_CHL_ = 84.2% for the 2014–2021 period), indicate a positive association. Another example is PEN and CEF (a third-generation cephalosporin), which belong to a large family of AMDs known as beta-lactams, also showed a positive association in the networks (e.g., R_PEN_|R_CEF_ = 77.4% and S_PEN_|S_CEF_ = 90.5% for 2014–2021). In contrast, aminoglycosides like GEN and NEO, along with their close relative SPC, an aminocyclitol, did not display direct links between them in any network. Resistance to aminoglycosides/aminocyclitols can occur via several mechanisms, such as enzymatic modification and inactivation of the aminoglycosides, increased efflux, decreased permeability, and alterations of the 30S ribosomal subunit that impedes aminoglycosides binding [5,23,24]. The lack of cross-resistance between these three aminoglycoside-aminocyclitol AMDs could be simply due to differences in their resistance mechanisms [23–25]. Additionally, tetracyclines, such as OXY and CHL, were not connected to TET, another member of the tetracycline family, in the networks, which is likely due to differences in MIC reporting practices: OXY and CHL data were reported throughout the entire study period, whereas TET data were only reported from 2018 onward. Nevertheless, the use of different AMDs within the same drug family may increase the risk of cross-resistance through shared resistance mechanism [26–28] and should therefore be approached with caution.

PEN and CEF are two effective and commonly used beta-lactams against *S. suis* infection in humans and pigs worldwide [17,29]. Despite the long-term use of beta-lactams in pigs, *S. suis* resistance to the members of this drug group is historically low [16]. We observed similar findings where *S. suis* resistance against PEN and CEF were 11% (n = 28) and 4.2% (n = 11), respectively. According to previous studies, this result may be due to an unusual mechanism used by *S. suis* to acquire resistance against beta-lactams, which is the modification of penicillin-binding proteins (PBPs) that are essential for peptidoglycan biosynthesis (either changed molecular weight, reduced affinity for beta-lactams, or both) [16].

Within the BNA, degree centrality quantifies the connectivity of an AMD node, corresponding to the number of direct conditional dependencies it holds with other AMD nodes [30]. PEN, TIA, and TIL were the most central nodes, with the highest degree of centrality in the overall network (2014–2021); PEN and TIA were most central in the 2019–2021 network, whereas TIL was most central in the 2014–2018 network. This centrality indicates that resistance to PEN, TIA, and TIL is highly associated with resistant outcomes of other AMDs. For example, in the 2019–2021 network, PEN was associated with the AMR classification of NEO, GEN, CEF, TIA, and SUL, while in the overall network (2014–2021), PEN was associated with SUL, TIA, GEN, and CEF. Given its central role in the network and its low resistance prevalence, PEN is particularly important in swine health and AMR control. Recent evidence has shown an increased emergence of *S. suis* resistance to beta-lactams, particularly PEN and cephalosporins like CEF, in certain regions [16,31–33], which may be attributed to their longstanding administration. Previous studies have identified specific point mutations within the transpeptidase domains of PBPs, such as PBP1a, PBP2a, PBP2b, and PBP2x, as primary resistance determinants in streptococci against these beta-lactams [33,34], and these mutations can be transferred to other bacteria via HGT mechanisms. Hence, proper use of beta-lactams in pigs is highly recommended to avoid the emergence and the spread of beta-lactam-resistant *S. suis*.

The linkages between AMDs of different, unrelated classes, such as those connected to PEN, suggest resistance will impact not only the effectiveness of PEN and other beta-lactams but also potentially that of aminoglycosides/aminocyclitols, sulfonamides, cephalosporins, and pleuromutilin derivatives. The other examples of such associations were linkages of lincosamides with macrolides, pleuromutilin, and tetracyclines, between pleuromutilin and aminocyclitols, and between macrolides and sulfonamides. These associations between unrelated drugs may be explained by various clinically important phenomena, including cross-resistance, where a single resistance mechanism confers resistance to multiple AMDs (e.g., multidrug efflux pumps), and co-resistance, where physical linkage of multiple resistant determinants (i.e. ARGs), each coding for resistance to a single drug, on a same genetic element.

Cross-resistance is a paradigm often used to describe resistance between AMDs belonging to the same class (e.g., PEN–CEF in beta-lactams, CHL–OXY in tetracyclines) due to a shared molecular mechanism. Nevertheless, cross-resistance can also occur between unrelated drugs if they exploit similar resistance mechanisms. For instance, macrolides and lincosamides are structurally distinct AMDs but share a similar mechanism of action, which is frequently used to explain cross-resistance between unrelated AMDs [35]. Both classes bind to the 50S ribosomal subunit to inhibit bacterial protein synthesis and are frequently used to treat gram-positive bacterial infections (e.g., *Staphylococcus* sp., *Streptococcus* sp.) when resistant to PEN. Resistance to these AMDs may arise through ribosomal alteration, drug inactivation, and overexpression of efflux pumps [35]. Due to shared resistance mechanisms and overlapping targets, concurrent use of macrolides and lincosamides is generally discouraged, as it offers limited clinical benefit and may promote resistance. Here, we identified a statistical association between TIL and CLN with higher conditional probabilities for S_TIL_|S_CLN_ (98.6%), R_TIL_|R_CLN_ (84.2%), S_CLN_|S_TIL_ (64.5%), and R_CLN_|R_TIL_ (99.5%), likely suggesting the cross-resistance between them. The presence of genes such as *ermB*, which encode the erythromycin ribosome methylase (Erm) enzyme, may contribute to this cross-resistance by methylating the bacterial ribosome, leading to the MLS_B_ (macrolide-lincosamide-streptogramin B) phenotype [35].

Another explanation for associations between unrelated AMDs is that genes conferring AMR to different AMD classes are inclined to locate together in the genomes of isolates (co-resistance) [36,37]. According to previous studies, many ARGs of various AMDs have been identified as passenger genes carried on MGEs and are disseminated to other bacteria via HGT mechanisms [5,38]. It is considered a critical driving force in bacterial evolution, especially in AMR distribution, and is often evident in *S. suis* [5]. For example, *ermB* and *tet(O)* genes encoding MLS_B_ and tetracycline, respectively, often co-occur on Integrative and Conjugative Elements (ICEs) [39], while aminoglycoside resistance genes (e.g., *ant(6)-Ia*, *ant(*9*)-Ia*), lincosamide resistance genes (e.g., *lnuB*) and TIA resistance genes (e.g., *lsaE*) have been identified together in ICEs (e.g., ICESsu584) [37]. Therefore, we can speculate that the observed statistical association between CLN–TET [39], SPC–CLN–TIA [37], and TIL–OXY [39] could be due to these physical linkages of genes on the same MGE. Strong conditional probabilities between resistance outcomes of CLN–TET (R_CLN_|R_TET_ = 81.5%, R_TET_|R_CLN_ = 94.7%), SPC–TIA (R_TIA_|R_SPC_ = 67.6%), and CLN–TIA (R_CLN_|R_TIA_ = 89.6%) may further support this hypothesis. Moreover, some of these drug combinations are also known for their synergistic effects when administered together, which occurs when the combined effect of two AMDs is larger than the additive effect of each drug. For example, synergisms between pleuromutilin (e.g., TIA) and aminoglycosides (e.g., SPC) against *S. suis* [40] as well as between CLN and TET against staphylococci (a gram-positive bacteria) [41] were previously described. All these AMDs are bacteriostatic agents where TIA and CLN bind to bacterial 50S while SPC and TET bind to the 30S subunit of ribosomes to inhibit bacterial protein synthesis. Therefore, the synergy between the two AMDs in each pair could be because of their distinctive bacterial targets and/or more complex interactions that happen when combined [42]. Such synergy is the basis of combination therapy and has been effectively practiced in swine production [40,43]. However, two potential conflicting evolutionary outcomes of synergy are recognized in which the risk of MDR can be either increased (via cross-resistance mechanisms) or decreased (via collateral sensitivity) in a given treatment, depending on infection properties such as mutation rate and the availability of resources [44–46].

Another example of a potential physical linkage is the association observed between beta-lactams (e.g., PEN) and pleuromutilin (e.g., TIA) with R_TIA_|R_PEN_ (71.2%), S_TIA_|S_PEN_ (87.1%), R_PEN_|R_TIA_ (37.2%), R_PEN_|S_TIA_ (4.1%), and S_PEN_|S_TIA_ (95.3%) conditional probabilities. According to a previous study, a *vgaF* homolog is often found upstream of the *pbp2b* gene [37], likely expressing resistance against pleuromutilin and beta-lactams, respectively, simultaneously. However, the genomic coordinates of ARGs were not explored in this study, thus we only speculate co-resistance as the mechanism for these observed associations. Therefore, further investigations are required to explore these genetic linkages.

Another potentially valuable relationship of beta-lactams in this study was with aminoglycosides/aminocyclitols such as GEN and SPC. We identified high conditional probabilities for S_GEN_|S_PEN_ (98.9%), S_SPC_|S_CEF_ (89.5%), S_PEN_|S_GEN_ (91.9%), and S_CEF_|S_SPC_ (98.5%), along with low conditional probabilities for R_GEN_|S_PEN_ (<1%), R_SPC_|S_CEF_ (10.1%), R_PEN_|S_GEN_ (7.2%), and R_CEF_|S_SPC_ (1.5%) indicating a strong positive association characterized by co-susceptibility between these AMDs. Nevertheless, conditional probabilities of S_GEN_|R_PEN_ (65.2%), S_SPC_|R_CEF_ (45.0%), S_PEN_|R_GEN_ (19.2%), and S_CEF_|R_SPC_ (86.5%) illustrate that PEN- or CEF-resistant isolates were not consistently resistant to aminoglycosides/aminocyclitols, and vice versa, indicating a lack of positive association in these resistance profiles. A previous study also described a negative correlation between PEN and aminoglycosides, such as GEN (−0.18, p < 0.01) and NEO (−0.22, p < 0.001) using a pairwise correlation analysis [47]. Regardless, based on the available literature, we cannot stipulate cross-resistance or co-resistance between these drugs; therefore, the underlying mechanisms of these associations remain unclear and require further investigation. Nevertheless, a robust synergistic effect between beta-lactams and aminoglycosides/aminocyclitols has been thoroughly discussed in previous studies [40,48–50]. According to these studies, the ability of beta-lactams to inhibit cell-wall synthesis by binding to PBPs may increase the permeation of aminoglycosides/aminocyclitols into the bacterial cytoplasm. As a result, beta-lactams may lower the MICs of aminoglycosides/aminocyclitols, likely reporting both AMDs within susceptible MIC ranges, in which case beta-lactams may be administrated before them [40]. Previous research has also demonstrated that combination therapy using beta-lactams (e.g., PEN, ampicillin) and aminoglycosides (e.g., GEN, apramycin) has been effective against various MDR pathogens, including *S. pneumoniae*, *S. agalactiae,* and *S. suis* [40,51–53]. However, it is important to note that clinical evidence regarding the superiority of β-lactam–aminoglycoside combination therapy over β-lactam monotherapy remains conflicting, despite promising in vitro and in vivo data [54].

In summary, our study captured various clinically meaningful AMR patterns of *S. suis* using BNA. Specifically, we demonstrated the complexity of AMR in *S. suis* by disclosing associations between AMDs across different drug classes. We portrayed not only pairwise relationships but also complex associations between multiple AMDs with relevant conditional probabilities. These relationships entail systematic dependencies between AMDs and may reflect underlying molecular mechanisms, such as cross-resistance or co-resistance at the genomic level. However, not all associations identified in this study can be explained based on current knowledge, and thus our findings are hypothesis-generating. Identifying various AMR patterns is particularly imperative in AMR epidemiology research, such as risk factor analysis, to subsequently examine causal determinants (e.g., farm factors, environmental factors, biosecurity measures) that drive the emergence and spread of these unique patterns. Further, our approach will serve as a valuable epidemiological tool in public and animal health, potentially leading to better use of AMDs to avoid lack of efficacy and overuse of drugs that could lead to resistance elsewhere.

We conducted this analysis using distinct production systems, which share a common source for the needs of resources such as gilts. Therefore, the endemic disease profiles of these farms are often similar. However, these endemic profiles are constantly challenged in response to various pressures (e.g., disease pressure, geographic location, and management), coercing AMR emergence and spread in varying degrees. Overuse of different antimicrobial compounds could induce more variation of AMR in *S. suis*, which may lead to complications in the treatment and control strategies. Therefore, the proper use of AMDs for prophylaxis and treatment in swine production systems is highly endorsed. Antibiotic combination therapy with distinct mechanisms of action offers a significantly more effective approach to treating MDR pathogens like *S. suis* and can help mitigate the development of resistance commonly associated with monotherapy [50]. Further, there is an increased oversight of antimicrobial uses in swine production systems globally. Thus, our findings raise awareness of the need for continuous surveillance of the susceptibility patterns of *S. suis* to assist and enhance antimicrobial stewardship.

Several limitations should be considered when interpreting these results. This study relied on passive syndromic surveillance data derived from routine clinical diagnostic submissions, introducing potential surveillance bias. Because most pigs are naturally colonized by *S. suis* without clinical disease, the AST data may not fully reflect the AMR profile of the broader *S. suis* population and may limit interpretation and generalizability to clinically affected pigs. In addition, detailed farm-level metadata, including herd characteristics, management practices, biosecurity measures, and AMD use, were not consistently available, limiting our ability to account for potential confounding factors. In particular, individual treatment histories, such as the timing of sample collection relative to AMD administration and whether treatment decisions were guided by prior antibiograms, were inconsistently reported. Consequently, clinical samples may represent a mixture of pre-treatment, post-treatment, or treatment-failure scenarios, and AST data should therefore be interpreted as reflective of routine diagnostic submissions rather than controlled treatment-response studies. Future studies incorporating systematic sampling, comprehensive farm-level data, and longitudinal treatment records could better characterize resistance dynamics and reduce surveillance and confounding biases. Additionally, interpretive MIC breakpoints and testing practices change over time; therefore, the findings from this paper may not directly translate to other studies using data from laboratories with different testing practices or from different time periods [55].

Despite these limitations, BNA was applied as a proof-of-concept to explore relationships among phenotypic resistance across multiple AMDs. While it does not establish causal relationships, BNA provides a framework to identify potential interactions among resistance traits and demonstrates the feasibility of applying this approach to AMR surveillance, informing future studies of resistance dynamics in swine populations. As this study focused exclusively on phenotypic resistance patterns, future studies integrating genomic analyses with BNA models may elucidate the underlying resistance mechanisms. Although no direct evidence of HGT was identified among the isolates analyzed here, previous studies have reported ARGs in *S. suis* located on MGEs, including integrative and conjugative elements and transposons, highlighting the potential for dissemination of AMR traits within *S. suis* populations [5,56,57].

Finally, intermediate interpretation is used in AMR classification when an AMD concentration is associated with an uncertain therapeutic effect in vitro. In practice, intermediate results are often grouped with either resistant or susceptible categories. However, according to the Clinical and Laboratory Standards Institute (CLSI), AMDs classified as intermediate may achieve treatment success (susceptible) if the infection is restricted to a site where the drug concentrates or when higher than standard dosages can be safely administered [58]. Thus, we grouped intermediate interpretations with susceptible readings to provide a conservative estimate of resistance profiles. However, this approach may not be appropriate for AMDs with a single approved dosing regimen or for drugs that do not concentrate at the site of infection. Further, intermediate resistance may signal the emergence of clinically relevant resistance; in such cases, grouping intermediate results with resistance may better reflect the risk of future resistance escalation. To assess the implications of our coding choice, we conducted a sensitivity analysis grouping intermediate interpretations with resistant. This reclassification slightly altered the network structure, eliminating three edges (PEN–GEN, OXY–TIL, and TIL–SUL), while introducing new edges, such as GEN–NEO, CHL–TIL, CHL–CLN, and CLN–SUL (S2 Fig). These changes reflect the conditional nature of Bayesian networks: edges represent dependencies that persist after conditioning on other variables, so a strong marginal association, such as the PEN–GEN edge in the original analysis, may no longer appear if reclassification shifts how shared variation is distributed across the network. Under the revised coding, GEN showed a stronger conditional alignment with NEO instead of PEN, separating off from the main connected component. Importantly, the overall network structure remained largely consistent across both analyses, with most key relationships preserved, providing confidence that the primary findings are robust to alternate classifications of intermediate results. However, this consistency is likely attributable to the low number of intermediate samples in the study.

## Conclusions

BNAs can be used to elucidate complex relationships between AMDs. The current study findings can be beneficial in generating hypotheses regarding animal and farm-level practices that may be associated with AMR resistance profiles. Future research could leverage AMD groupings identified in BNA as dependent variables in regression or risk factor analyses to identify factors that explain the occurrence of resistance profiles. Additionally, BNAs provide a useful and visual way to explore phenotypic resistance profiles and associations among bacterial isolates. If combined with other animal and farm-level information, the resulting graphs could be beneficial in making management and treatment recommendations.

## Methods

### Data collection

This study was conducted as a secondary analysis using existing culture and antimicrobial susceptibility data obtained through passive syndromic surveillance. The analysis included data from 259 unique *S. suis* isolates collected from clinically infected pigs in 91 swine farms representing different production systems in the United States between 2014 and 2021. All samples originated from routine clinical diagnostic submissions and were processed at the ISU-VDL for *S. suis* isolation, identification, and antimicrobial susceptibility testing against a panel of antimicrobials. The dataset did not include animal-level or farm-level risk factor information, and therefore such variables could not be incorporated into the analysis.

### Antimicrobial susceptibility testing (AST)

Phenotypic susceptibility and resistant data were generated from *S. suis* isolates by ISU-VDL following previously described protocols [59]. Briefly, in-vitro ASTs were conducted for 16 AMDs. The minimum inhibitory concentrations (MICs) of each strain were determined by the broth microdilution method using the Sensititre BOPO7F plates and the Sensititre system (Trek Diagnostic Systems, Cleveland, Ohio) [59], and ASTs were performed according to an interpretation report received from the American Association of Veterinary Laboratory Diagnosticians (AAVLD) accredited laboratory in the United States. These MIC interpretations were used for BNA to identify joint patterns of resistance (resistance profiles). *S. suis* strains were classified as susceptible, intermediate, or resistant to each AMD (S1 Table). MICs interpreted as intermediate (n = 73, 2.5%) were grouped with susceptible to create a binary phenotypic variable of resistant or susceptible. The AMDs ampicillin, florfenicol, and trimethoprim-sulfamethoxazole were removed from the analysis because less than ten isolates showed phenotypic resistance for each. This left MIC interpretations for ceftiofur (CEF), penicillin (PEN), enrofloxacin (ENR), gentamicin (GEN), neomycin (NEO), spectinomycin (SPC), sulfadimethoxine (SUL), tiamulin (TIA), tilmicosin (TIL), clindamycin (CLN), chlortetracycline (CHL), oxytetracycline (OXY), and tetracycline (TET) in the analysis.

### Bayesian network analysis (BNA)

Bayesian networks (BNs), presented as directed acyclic graphs (DAGs), represent a joint probability distribution and describe probabilistic dependencies between a set of variables [13]. BNs consist of nodes representing the variables under study and edges representing associations between the variables. There are three distinct parts to BNA, as previously described [12]. Briefly, this analysis consisted of network learning (i.e., identifying the topological relationships between nodes), a bootstrap analysis with 10,000 iterations to identify the most consistent connections and remove potentially spurious associations, and parameter learning to determine the conditional probabilities between nodes. In the bootstrap step, each resampled dataset undergoes the same score-based learning procedure using BIC to produce a separate network structure. The *boot.strength* function then quantifies the stability of each arch by estimating its strength, defined as the frequency with which that arc appears across the 10,000 bootstrap networks. These strengths are subsequently used to construct a consensus network, defined as the set of arcs with strength greater than 50%, representing the most robust and consistently supported connections maintained in the final graph.

We used a purely data-driven, exploratory approach to model the relationships between MIC interpretation variables. To account for clustering of observations within farms, we treated the farm identifier variable as a higher-level node and used tiered blocklist constraints to prohibit lower-level variables from being parents of farm, allowing the network to capture site-level structure without forcing specific edges [60]. The score-based structure learning algorithm, Tabu, was used to identify the network structure. Tabu is a modified greedy hill-climbing search that uses goodness-of-fit scores as objective functions to find the global optimum [61]. Because the network is discrete, conditional independence between the nodes was estimated using a log-likelihood ratio test [62].

The expectation-maximization (EM) algorithm, an iterative procedure computing the expected sufficient statistics conditional on the observed data using belief propagation, was used to smooth over missing values [63]. We used the expectation-maximization algorithm to test both the parents’ and Bayes’ imputation methods. The parents’ imputation computes predicted values by plugging in new values in the local probability distribution for the parent node. Bayes’ imputation computes predicted values by averaging likelihood weighting simulations using all the nodes as evidence [64]. Parameter learning was conducted using the Bayes’ method. This method uses the expected values of the parameters’ posterior distribution arising from a flat prior and is less prone to overfitting than maximum likelihood estimation [65]. For missing values, the Bayes’ method estimates the parameters of each local distribution using complete observations for the nodes and parents involved [64]. We built an overall network using the steps outlined above. While *bnlearn* produces DAGs, due to the cross-sectional nature of the data and the inability to determine temporality in AMD resistance, causality was not inferred from the data. Therefore, the arc directions for the DAGs in the manuscript were removed for simplicity of interpretation. The original, unaltered DAGs can be viewed in S3 Fig.

While OXY and CHL data were available throughout the entire study period (2014–2021), TET data only became available from 2018 onward due to changes in MIC reporting practices. Accordingly, TET data were limited during 2014–2018 (13/119), whereas OXY and CHL data were available for most isolates (106/119). In contrast, during 2019–2021, TET data were available for most isolates (131/140), while OXY and CHL data were reported for only a small subset (9/140). To explore how different tetracyclines (TET, OXY, and CHL) related to other AMDs under varying data availability conditions, the dataset was divided into two periods: 2014–2018 and 2019–2021. These timeframes were selected to reflect shifts in tetracycline data availability while also maintaining a relatively balanced number of isolates: 119 (45.9%) and 140 (54.1%), respectively. Subnetworks were constructed for each period using the same analytical approach as for the overall network. These period-specific subnetworks were intended to examine associations under differing data availability conditions and were not designed to assess or compare temporal changes in resistance patterns. All analyses were conducted in R (version 4.4.1) using the Bayesian network learning package *bnlearn* [64].

## Supporting information

S1 Table The MIC breakpoints used to assign *S. suis* isolates to interpretative susceptibility categories. (PDF)

S2 Table Conditional probability table for the overall network. (XLSX)

S1 Fig AMR patterns among *S. suis* isolates. (PDF)

S2 Fig Sensitivity analysis network with the intermediate category grouped with resistance. (PDF)

S3 Fig Original, unaltered DAGs of the three networks. (PDF)
