# Correction: Bayesian network models to assess antimicrobial resistance patterns of *Streptococcus suis* isolated from swine production systems in the United States between 2014–2021

**DOI:** 10.1371/journal.pcbi.1014345

**Published:** 2026-05-28

**Authors:** Ruwini Rupasinghe, Brittany L. Morgan Bustamante, Rebecca C. Robbins, Maria J. Clavijo, Beatriz Martínez-López

The last two authors, Maria J. Clavijo and Beatriz Martínez-López, should be noted as shared co-corresponding authors on this work. Maria J. Clavijo’s email address is: mclavijo@iastate.edu.
