# Supplementary material for: Bayesian network models to assess antimicrobial resistance patterns of Streptococcus suis isolated from swine production systems in the United States between 2014–2021
Source: PLoS Comput Biol. 2026 Mar 26;22(3):e1014117. doi: 10.1371/journal.pcbi.1014117 (PMC13020804; doi:10.1371/journal.pcbi.1014117)
Supplement: S1 Table — (PDF) [file pcbi.1014117.s001.pdf]

S1 Table. The Minimum Inhibitory Concentration (MIC) breakpoints used to assign *Streptococcus suis* isolates to interpretative susceptibility categories: susceptible (S), intermediate (I), and resistant (R) (ND: not defined).

**Antimicrobial drug**	**MIC breakpoints (µg/mL)**		
	**S**	**I**	**R**
Ampicillin	≤ 0.5	1	≥ 2
Ceftiofur	≤ 2	4	≥ 8
Penicillin	≤ 0.25	0.5	≥ 1
Chlortetracycline	<= 0.5	1	>= 2
Oxytetracycline	<= 0.5	1	>= 2
Tetracycline	≤ 0.5	1	≥ 2
Tilmicosin	≤ 16	ND	> 16
Enrofloxacin	≤ 0.5	1	≥ 2
Gentamicin	≤ 4	8	≥ 16
Neomycin	≤ 8	ND	> 8
Spectinomycin	≤ 64	ND	≥ 128
Sulfadimethoxine	<= 256	ND	> 256
Trimethoprim/sulfamethoxazole	≤ 2	ND	> 2
Clindamycin	≤ 0.25	0.5	≥ 1
Florfenicol	≤ 2	4	≥ 8
Tiamulin	≤ 16	ND	>16
