# Supplementary material for: Bayesian network models to assess antimicrobial resistance patterns of Streptococcus suis isolated from swine production systems in the United States between 2014–2021
Source: PLoS Comput Biol. 2026 Mar 26;22(3):e1014117. doi: 10.1371/journal.pcbi.1014117 (PMC13020804; doi:10.1371/journal.pcbi.1014117)
Supplement: S1 Fig — (PDF) [file pcbi.1014117.s003.pdf]

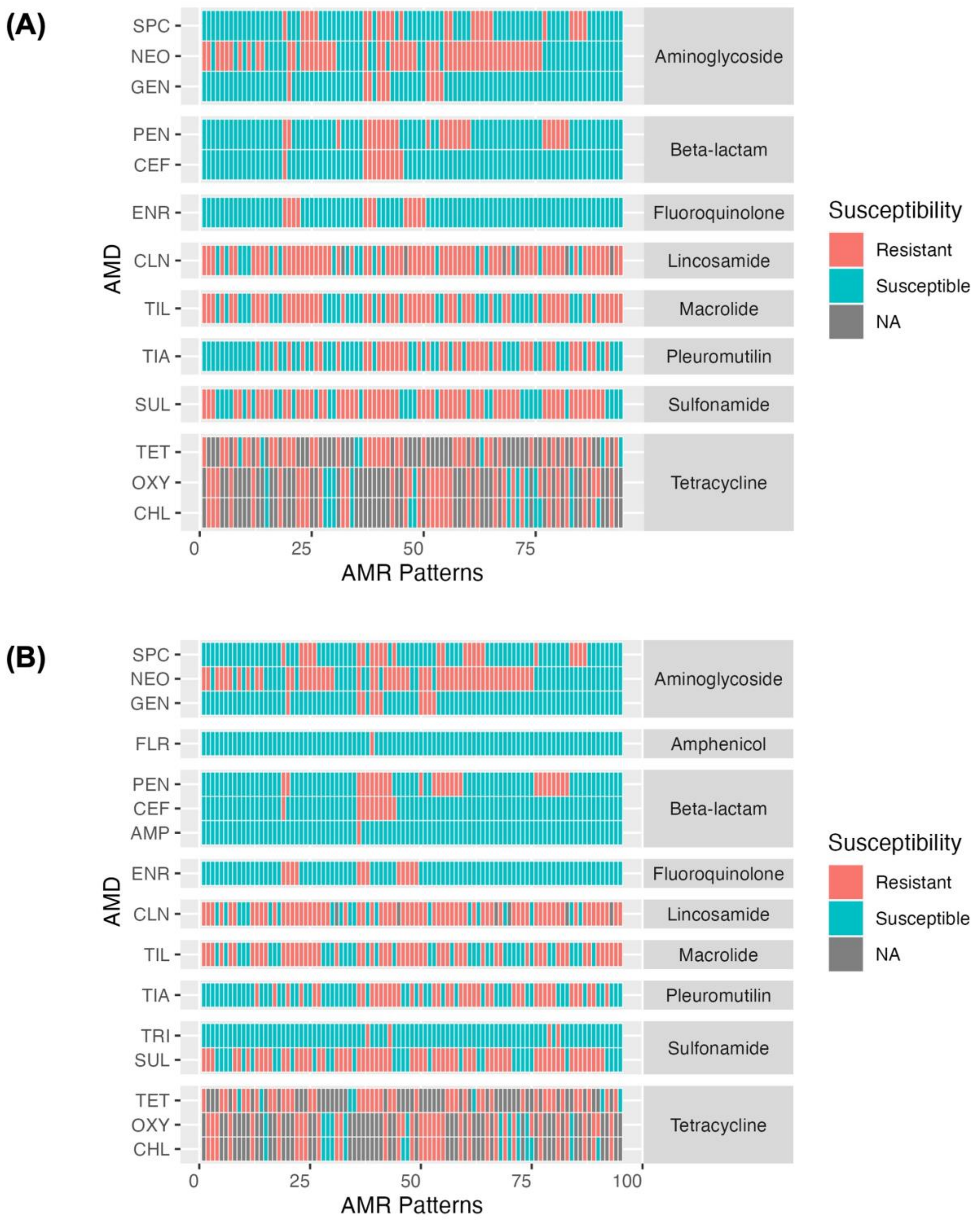

S1 Fig. Antimicrobial resistance (AMR) patterns among 259 unique *Streptococcus suis* isolates collected from clinically infected pigs (2014-2021). MICs interpreted as intermediate

were grouped with susceptible for each antimicrobial drug (AMD). **(A)** AMR patterns based on AMDs included in the BNA analysis, with 94 distinct patterns observed. **(B)** AMR patterns based on all AMDs with available MIC interpretations, including those omitted from the analysis due to a low number of resistant isolates, with 95 distinct patterns observed. NA indicates isolates with no MIC information for the respective AMDs. AMDs included spectinomycin (SPC), neomycin (NEO), gentamicin (GEN), florfenicol (FLR), penicillin (PEN), ceftiofur (CEF), ampicillin (AMP), enrofloxacin (ENR), clindamycin (CLN), tilmicosin (TIL), tiamulin (TIA), trimethoprim-sulfamethoxazole (TRI), sulfadimethoxine (SUL), tetracycline (TET), oxytetracycline (OXY), and chlortetracycline (CHL).
