# Supplementary material for: Bayesian network models to assess antimicrobial resistance patterns of Streptococcus suis isolated from swine production systems in the United States between 2014–2021
Source: PLoS Comput Biol. 2026 Mar 26;22(3):e1014117. doi: 10.1371/journal.pcbi.1014117 (PMC13020804; doi:10.1371/journal.pcbi.1014117)
Supplement: S2 Fig — (PDF) [file pcbi.1014117.s004.pdf]

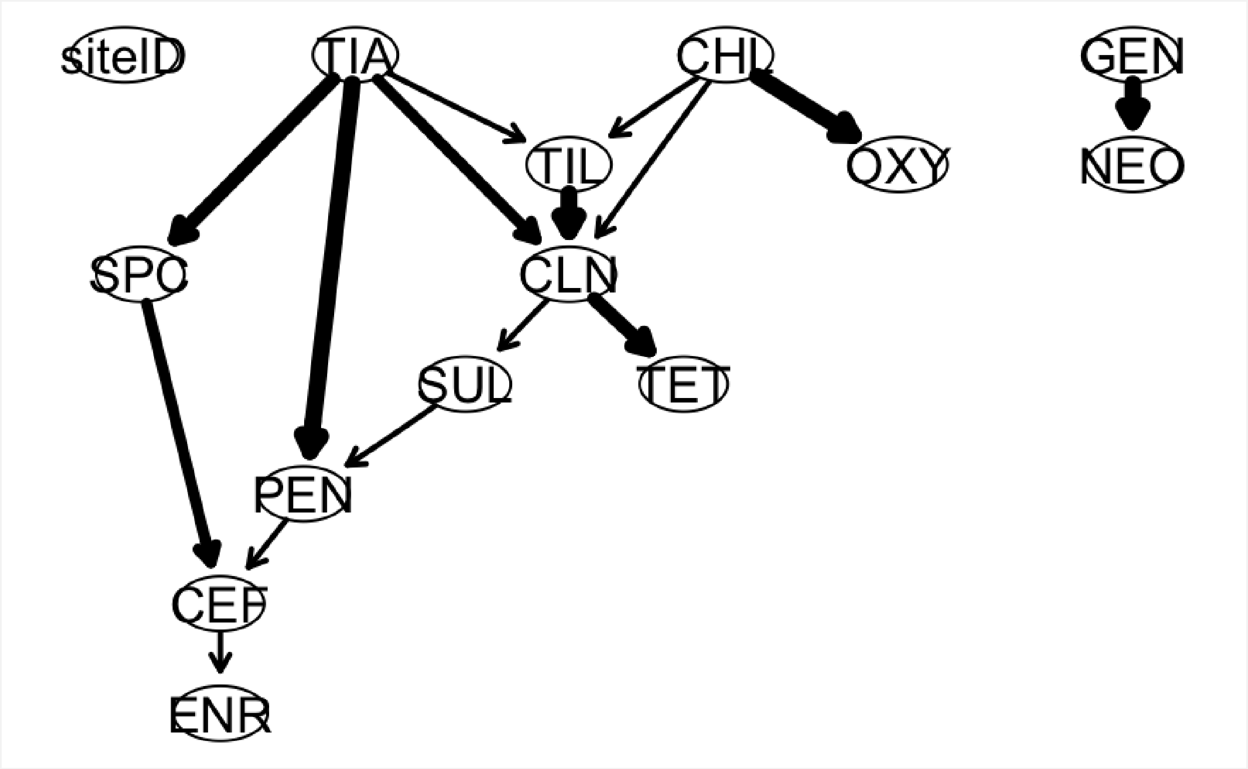

S2 Fig. Sensitivity analysis network with the intermediate category grouped with resistance (2014-2021). Original, unaltered directed acyclic graphs (DAGs) generated using *bnlearn* depict relationships among phenotypic *Streptococcus suis* resistance classifications (resistant vs. susceptible, with intermediate categorized as resistant) across antimicrobial drugs (AMDs) for isolates obtained from swine during 2014-2021. Edge thickness reflects bootstrap arc strength, defined as the frequency with which each arc was recovered across 10,000 bootstrap networks; only arcs with bootstrap strength >50% are shown. The farm identifier (siteID) is a contextual variable used to account for clustering of isolates by production site. Nodes represent AMDs: Ceftiofur (CEF), enrofloxacin (ENR), gentamicin (GEN), neomycin (NEO), penicillin (PEN), spectinomycin (SPC), sulfadimethoxine (SUL), tiamulin (TIA), tilmicosin (TIL), chlortetracycline (CHL), clindamycin (CLN), oxytetracycline (OXY), and tetracycline (TET).
