# Supplementary material for: Bayesian network models to assess antimicrobial resistance patterns of Streptococcus suis isolated from swine production systems in the United States between 2014–2021
Source: PLoS Comput Biol. 2026 Mar 26;22(3):e1014117. doi: 10.1371/journal.pcbi.1014117 (PMC13020804; doi:10.1371/journal.pcbi.1014117)
Supplement: S3 Fig — (PDF) [file pcbi.1014117.s005.pdf]

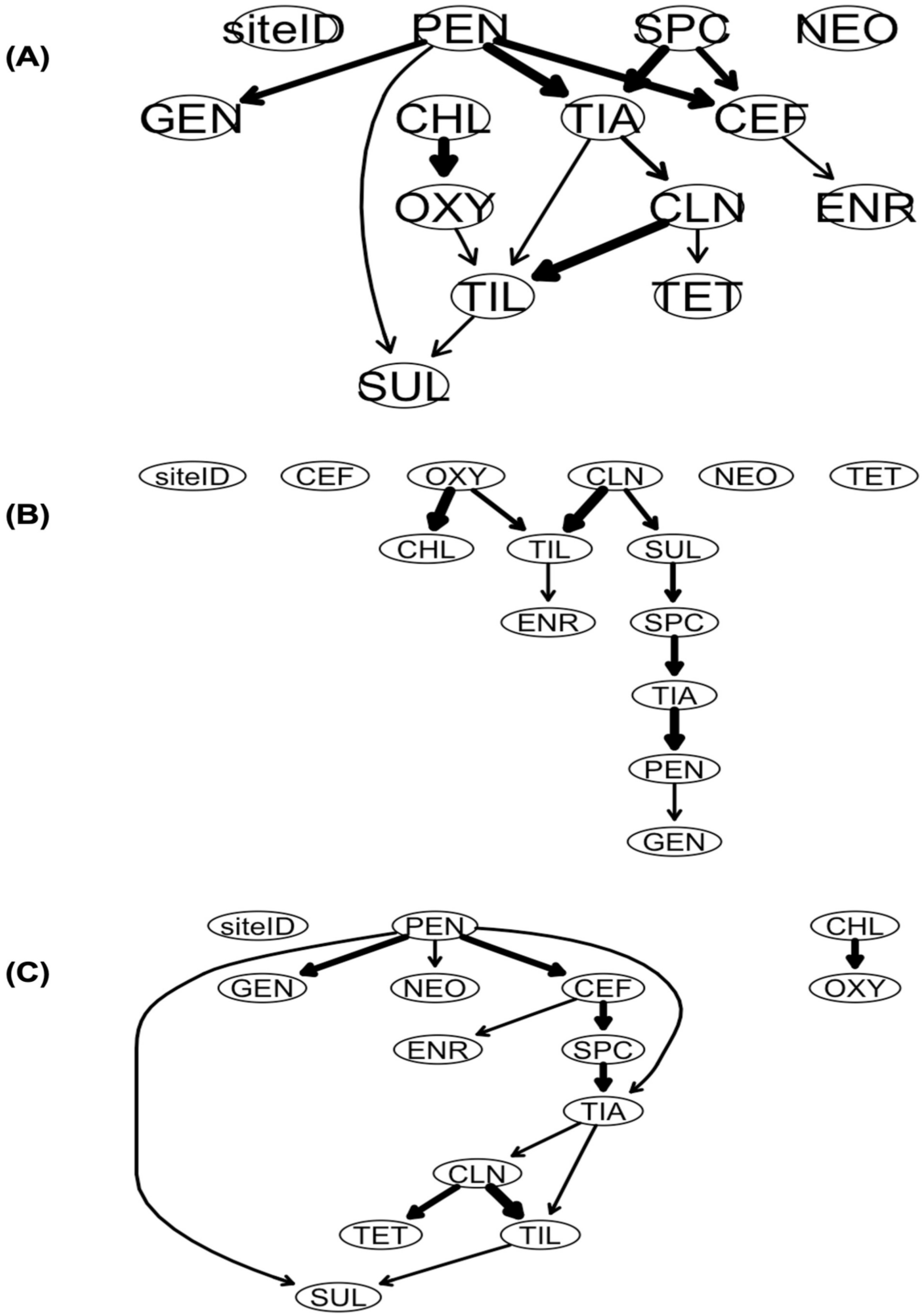
(A)
siteID
PEN
SPC
NEO
GEN
CHL
TIA
CEF
OXY
CLN
ENR
TIL
TET
SUL
(B)
siteID
CEF
OXY
CLN
NEO
TET
CHL
TIL
SUL
ENR
SPC
TIA
PEN
GEN
(C)
siteID
PEN
CHL
GEN
NEO
CEF
OXY
ENR
SPC
TIA
CLN
TET
TIL
SUL

S3 Fig. Original, unaltered directed acyclic graphs (DAGs) generated using *bnlearn* showing relationships among phenotypic *Streptococcus suis* resistance classifications. Linkages represent associations between resistance classifications (resistant *vs.* susceptible, with intermediate categorized as susceptible) across antimicrobial drugs (AMDs) for isolates obtained from swine during **(A)** 2014-2021, **(B)** 2014-2018, and **(C)** 2019-2021. Edge thickness reflects the bootstrap arc strength, defined as the frequency with which each arc was recovered across 10,000 bootstrap networks; only arcs with bootstrap strength >50% are shown. The farm identifier (siteID) is a contextual variable used to account for clustering of isolates by production site. Nodes represent AMDs: Ceftiofur (CEF), enrofloxacin (ENR), gentamicin (GEN), neomycin (NEO), penicillin (PEN), spectinomycin (SPC), sulfadimethoxine (SUL), tiamulin (TIA), tilmicosin (TIL), chlortetracycline (CHL), clindamycin (CLN), oxytetracycline (OXY), and tetracycline (TET).
